# Stability and Rheological Properties of Grouts with Waste Glass Powder as Cement Replacement: Influences of Content and Alkali Activator

**DOI:** 10.3390/ma18020353

**Published:** 2025-01-14

**Authors:** Liuxi Li, Chao Deng, Yi Zhou, Qundong Tan, Wenqin Yan, Dequan Zhou, Yi Zhou

**Affiliations:** 1 College of Civil Engineering, Changsha University of Science and Technology, Changsha 410114, China; liliuxi@hncu.edu.cn (L.L.); zhoudequan28@csust.edu.cn (D.Z.); 2 Hunan Engineering Research Center of Structural Safety and Disaster Prevention for Urban Underground Infrastructure, Hunan City University, Yiyang 413000, China; zhouyi@hncu.edu.cn; 3 College of Civil Engineering, Hunan City University, Yiyang 413000, China; 13272376205@163.com; 4 School of Geosciences and Info-Physics, Central South University, Changsha 410083, China; 235001037@csu.edu.cn

**Keywords:** sustainable construction materials, glass waste valorization, cementitious replacement, alkaline activation, grouting properties

## Abstract

Effective recycling and utilization of waste glass is a critical issue that urgently needs to be addressed. This study aims to explore the feasibility of using ground waste glass powder (particle size ≤ 75 μm) as a supplementary cementitious material to partially replace cement in the preparation of low-carbon and environmentally friendly grouting materials. The research systematically evaluates the impact of waste glass powder (WGP) on the fresh properties (particularly the stability and rheological characteristics) of cement-based grouting materials under various conditions, including WGP content (0–40%), the addition of NaOH activator (Na_2_O content of 4%) or not, and water–solid ratio (*w*/*s =* 0.5, 0.65, 0.8, 1.0). The results indicate that, in the absence of activator, the addition of WGP generally increases the amount of free liquid exudation in the grout, reducing its stability; however, under low *w*/*s* ratios, appropriate amounts of WGP can enhance stability. When the *w*/*s* ratio is high and the WGP content is large, the grout stability decreases significantly. The addition of NaOH activator (Na_2_O content of 4%) significantly reduces free liquid exudation, enhancing the stability of the grout, especially when the *w*/*s* ratio is less than 1.0. Furthermore, the Herschel–Bulkley Model was experimentally validated to accurately describe the rheological behavior of waste glass–cement slurries, with all *R*^2^ values exceeding 0.99. WGP and alkaline activator have significant effects on the rheological properties of the grout. Although they do not change its flow pattern, they significantly affect shear stress and viscosity. The viscosity of the slurry is influenced by the combined effects of *w*/*s* ratio, WGP content, and alkaline activator, with complex interactions among the three. The application of these research findings in the field of grouting engineering not only contributes to significantly reducing glass waste but also promotes the production of sustainable cement-based composites, lowering carbon dioxide emissions by reducing cement usage, and thereby alleviating environmental burdens.

## 1. Introduction

In the “Global Waste Management Outlook 2024” report released by the United Nations, it is stated that the global production of urban solid waste is expected to increase from 2.3 billion tons in 2023 to 3.8 billion tons in 2050. Glass, one of the earliest materials invented by humans, has led to the generation of approximately 100 million tons of waste glass annually due to the widespread use of glass products amidst the acceleration of global industrialization [1], accounting for about 5% of the total global solid waste [2]. Therefore, the issue of waste glass, as a ubiquitous solid waste, has become an increasingly significant focal point for attention in terms of its treatment and resource utilization.

The primary methods for disposing of waste glass are landfilling and incineration [1,2], with only a small portion being recycled and reused in China. Landfilling not only consumes a significant amount of land resources but also poses potential risks of soil and groundwater contamination. Incineration, on the other hand, faces challenges during processing and emits a substantial amount of harmful gases such as CO_2_, contributing to secondary environmental pollution. Consequently, the resourceful utilization of waste glass has emerged as a crucial solution to address the challenges associated with its disposal. The utilization of waste glass includes direct reuse, utilization as raw material for glass production, and application in construction materials [3]. However, due to the difficulties in recycling waste glass, limited options, and low economic benefits, its recycling rate remains generally low in China (according to China’s Recycling Industry Development Report 2024). Therefore, effectively utilizing waste glass resources, enhancing their recycling rate, and creating economic value are important issues that urgently need to be addressed.

Grouting cementitious materials, as vital components in engineering, are extensively applied in fields such as foundation reinforcement, waterproofing and leakage plugging, lifting and rectification, tunnel support, and mine filling [4,5]. Traditional grouting cementitious materials are primarily cement-based. The cement industry accounts for 8% of global anthropogenic carbon dioxide emissions [6], and it is projected that global cement consumption will double by 2050 [7]. Cement-based grouting materials aim to improve grout performance, expand engineering applications, and enhance grouting effects, while adhering to the concept of green and sustainable economic development, and moving towards the development of eco-friendly and high-performance grouting cementitious materials [8]. Consequently, the search for an environmentally friendly, energy-efficient, and resource-recycled grouting cementitious material to replace traditional cement has become an important research direction with regard to grouting materials. Glass, a non-crystalline inorganic non-metallic material, exhibits excellent chemical stability and strength [9]. It is mainly composed of silicon dioxide (SiO_2_), sodium oxide (Na_2_O), calcium oxide (CaO), and other components, possessing potential pozzolanic activity and serving as a potential substitute for cementitious materials [10,11]. In the field of grouting engineering, researchers such as Güllü [12] have attempted to apply waste glass by using cement-based grouting materials mixed with waste glass powder (WGP) to enhance clayey soil foundations through deep mixing techniques. Tho-In et al. [13] investigated the compressive strength and microstructure of geopolymer grouts containing WGP and high-calcium fly ash. The resourceful utilization of waste glass in grouting engineering not only addresses waste disposal issues but also reduces dependence on traditional cement, thereby alleviating resource scarcity and mitigating environmental pollution.

The research and application of waste glass in concrete have yielded fruitful results, with waste glass being used as a substitute for coarse aggregates [14,15,16,17], fine aggregates [18,19,20], and cement [21]. By controlling factors such as mix proportions, particle size ranges, and curing environments, glass concrete with satisfactory performance and excellent properties can be produced [22,23,24]. Silicate glass, with silicon dioxide as its main component, is the most common form of glass waste, and its hardness is similar to that of sand and stone. Kuri et al. [25] partially replaced coarse aggregates with waste glass to prepare ordinary Portland cement concrete and alkali-activated geopolymer concrete, achieving excellent concrete performance with a 20% waste glass replacement ratio. However, when waste glass is incorporated as an aggregate in concrete, its high alkali content can easily induce the harmful alkali–silica reaction (ASR) [26,27,28]. The mechanical properties of glass concrete and ASR expansion are closely related to the particle size and replacement ratio of waste glass particles. Khan et al. [20] found that geopolymer mortar using glass fine aggregates did not exhibit any detrimental alkali–silica reactions. As the particle size of glass particles decreases, at the same replacement ratio, the mechanical properties significantly improve, and ASR expansion markedly reduces [29]. The use of WGP as a cementitious material in ordinary concrete has been extensively studied and widely applied [30]. Regardless of the glass type, the addition of WGP as a cement substitute can greatly enhance the workability of concrete mixtures. Finer WGP with a larger specific surface area facilitates sufficient pozzolanic activity [31,32]. Meanwhile, alkali activators such as sodium hydroxide and potassium hydroxide serve as effective chemical activators, enhancing the release and dissolution of active components in WGP, i.e., improving its early activity. In the presence of WGP, alkali-activated mortars and concretes often exhibit better mechanical properties [33], demonstrating great potential in promoting the resource utilization of industrial wastes like waste glass. In recent years, research on alkali activators has deepened, with significant progress made in aspects such as their types, dosages, and synergistic effects with other additives [2,34,35,36]. Waste glass rich in silicon dioxide, as a precursor for sustainable geopolymer binders, can combine with alumina-containing materials like slag, fly ash, or metakaolin to form the necessary components for geopolymerization [37]. These research findings not only provide a theoretical basis for the efficient utilization of waste glass in concrete but also open up new avenues for the development of novel eco-friendly grouting cementitious materials.

In summary, research on the application of waste glass in concrete and alkali-activated geopolymers has shown that stable waste glass contains a large amount of amorphous silicon dioxide [38], with a chemical composition similar to fly ash, exhibiting potential pozzolanic activity and thus can be used as a cement substitute [11,39,40]. This provides a solid theoretical foundation and practical guidance for the present study, as shown in Table 1, different combinations of waste glass with other by-products. This study aims to explore the potential application value of WGP as a grouting cementitious material. The specific objectives include exploring new application pathways for WGP as a grouting cementitious material to expand its application fields; systematically studying the influence of the amount of WGP and alkaline activator on the properties of freshly mixed grout to provide a theoretical basis for practical applications; achieving efficient utilization of waste glass resources by partially replacing cement, reducing dependence on traditional cement; and promoting sustainable resource development.

High stability and fluidity are fundamental performance requirements for cement-based grouts in various grouting engineering applications [5,8,41]. The improvement of the rheological and stability properties of grouts is a crucial aspect of the continuous advancement of grouting technology [42]. Furthermore, extensive studies on the rheological properties of cement grouts have demonstrated that these properties significantly impact the grouting effect. For instance, references [43,44,45,46] indicate that cement grouts should maintain stable bleeding and settlement characteristics, as excessive bleeding and unstable settlement can lead to incomplete filling during grouting, resulting in the loss of reinforcement and waterproofing effects. The rheological properties of cement-based grouts determine their ultimate diffusion within the geotechnical medium [46,47]. Additionally, the rheological parameters of cement grouts are of great significance for grouting design and the prediction of grouting effects [48], serving as prerequisites for effective grouting outcomes and designs [49].

**Figure 1:**
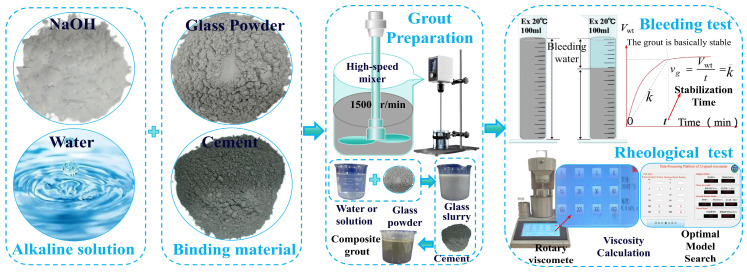
The schematic diagram of the implementation process for research.

The use of WGP as a supplementary cementitious material (SCM) is feasible and holds great potential for development. Mirzahosseini et al. [50] investigated the effects of glass powders with particle size distributions of 0–25 μm, 25–38 μm, and 63–75 μm on cement mortar, and found that mortar with particles in the 0–25 μm range exhibited the highest compressive strength. Therefore, utilizing milled WGP as an SCM to partially replace cement in the production of low-carbon cement represents an effective approach to promoting the efficient utilization of waste glass resources. The performance of waste glass composites is influenced by various factors such as the water-to-cement ratio, glass particle size, dosage, and curing age [51]. Although there have been reports on the performance of concrete incorporating cementitious materials composed of glass sand or glass powder, a consensus on their effects on concrete material performance has not yet been reached [52,53,54,55]. Currently, there is limited research on the impact of WGP on the basic grouting properties of cement-based grouting materials [56,57,58]. Therefore, this study focuses on using WGP as a partial cement replacement to prepare grouts with different water–solid ratios (*w*/*s* = 0.5–1.0). It systematically explores the influence of WGP content (0–40%) on the stability and rheological properties of WGP–cement-based grouts with and without the addition of NaOH activator at various *w*/*s* ratios, the implementation process of the research is illustrated in Figure 1. The study also analyzes the mechanism of waste glass powder’s impact on the stability and viscosity of cement grouts. This study aims to provide a novel approach for the application of waste glass in the field of grouting engineering, elucidate the effects of WGP on the stability and rheological properties of grout, and conduct a preliminary exploration of the use of waste glass in grouting applications.

## 2. Experimental Materials and Methods

### 2.1. Raw Materials

**Figure 2:**
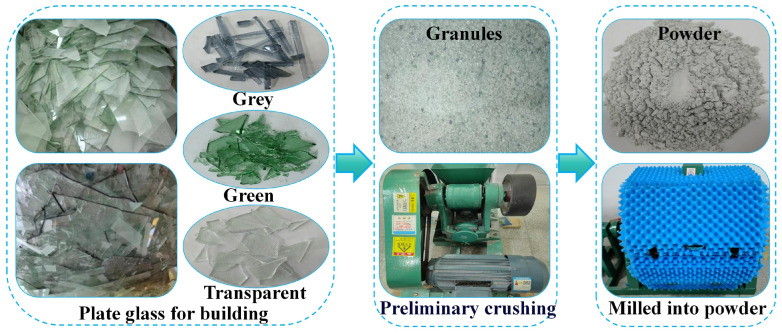
Preparation of WGP for the experiment.

The experimental procedure conducted in this study is illustrated in Figure 1, which clearly indicates that raw materials such as water, cement, waste glass, and alkaline activator will be utilized. For this experiment, P.O 42.5 ordinary Portland cement (Yiyang Shaofeng Cement Co., Ltd., Yiyang City, China) from the same production batch was selected as the cementitious base material for preparing the grout. Some of the performance indicators of this cement are shown in Table 2. The waste glass used in the experiment was sourced from recycled flat glass from construction waste, with a color distribution of transparent (up to 92%), green (6.3%), and gray (1.7%) colors. The impurities in the glass were removed, and it was washed and dried for later use. The glass was initially crushed to a particle size of approximately 2–6 mm using a jaw crusher, and then further ground in an SEM-500 small ball (Shaoxing City, China) mill to obtain WGP. By controlling the grinding time, WGP with different median particle sizes could be obtained. The process for obtaining the WGP used in the experiment is shown in Figure 2. The water used in the experiment was tap water from Hunan City University, which was allowed to stand in a storage tank for 48 h to settle impurities and remove chlorine. Since NaOH has a stronger ability to release silicate and aluminate monomers compared to other alkaline activators [59], AR-grade NaOH (CAS No. 1310-73-2) with a purity of ≥99.0%, in the form of flaky semi-transparent solids, was chosen as the alkaline activator for the experiment.

**Figure 3:**
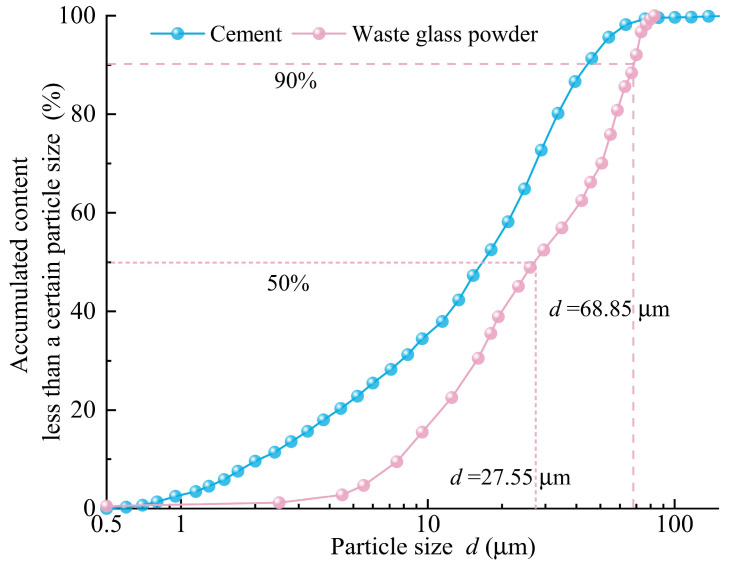
Gradation curve of the cement and WGP.

**Figure 4:**
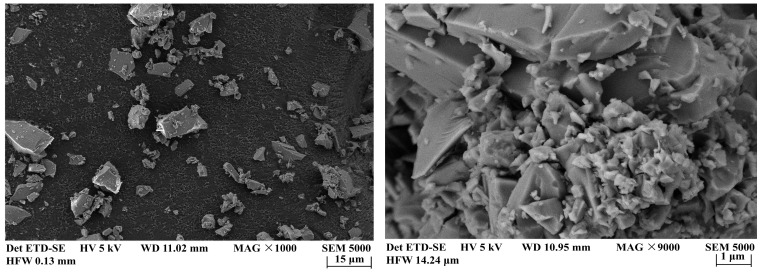
SEM image of WGP (magnification ×1000 and ×9000).

Previous studies have shown that as the fineness of glass powder increases, its pozzolanic activity is gradually enhanced [60]. Aliabdo et al. [61] found that by replacing 10% of cement with glass powder particles smaller than 75 μm, the compressive strength of mortar after 7 days can be increased by approximately 9.0%. Therefore, in this study, the WGP (with a density of 2.58 g/cm^3^, water absorption of 0.01, and loss on ignition of 1.1%) used was obtained by sieving WGP milled for 30 min through a 200-mesh screen. Waste glass particles larger than 75 μm were excluded to ensure the pozzolanic activity of the WGP. The particle size and chemical composition of the cement and WGP were tested using a Malvern 2000 laser particle size analyzer (Bruker, Leipzig, Germany) and a D8-Advance X-ray diffractometer (Bruker, Malvern, UK). The particle size distribution curves (Figure 3) and chemical compositions (Table 3) of the cement and WGP were analyzed. Figure 3 indicates that the median particle size of the WGP used in the experiment is *d*_50_ = 27.55 μm, and 90% of the WGPr particles have a size smaller than 68.85 μm (*d*_90_ = 68.85 μm). Clearly, compared to P.O 42.5 Portland cement, waste glass has a higher SiO_2_ content and a lower CaO content, with a particularly high Na_2_O content of 12.72% (as shown in Table 3), which is also the reason for the potential for alkali–silica reaction (ASR) that occurs when using waste glass as an aggregate in cement-based composites. SEM micrographs (as shown in Figure 4) show that the WGP particles used in the experiment have various shapes, significant differences in particle size, sharp edges, smooth surfaces, and small particles aggregated on larger ones to form clustered aggregates.

### 2.2. Comprehensive Experimental Design

To investigate the impact of WGP as a supplementary cementitious material on the basic grouting properties (stability, rheology) of cement grout, cement grouts with different water-to-cement ratios (*w*/c = 0.5, 0.65, 0.8, 1.0) without WGP were used as the control group. Tests were conducted to evaluate the performance of cement-based grouts with varying amounts of WGP under different water–solid (*w*/*s*) ratios, both with and without the addition of an alkali activator. Here, “solid” refers to the total mass of waste glass, cement, and sodium hydroxide solids. Numerous studies on waste glass–cement composites have indicated that the dosage of alkali activator is calculated based on the Na_2_O content. Zhang et al. [62,63] demonstrated that an alkali content (Na_2_O dosage as a percentage of the cementitious material mass) of 4% is optimal for the workability and physical–mechanical properties of cement-based composites. Therefore, in this study, the dosage of the alkali activator was determined, such that the Na_2_O content accounted for 4% of the cementitious material (calculated based on the mass of cement and waste glass). The actual dosage was calculated and fully dissolved in water 24 h before the experiment, allowing the NaOH solution to cool to laboratory ambient temperature for later use. The dosage of WGP was calculated as a percentage of the total cementitious material mass, with waste glass content of 0%, 5%, 10%, 20%, 30%, and 40%. The cement grout with a *w*/*s* ratio corresponding to 0% WGP content serves as the experimental control group. The specific experimental mix proportions are shown in Table 4. A comprehensive experimental program was conducted to test the performance of waste glass–cement-based grouts under different mix proportions, requiring the evaluation of 44 different groups of cement grout performance tests.

### 2.3. Sample Preparation

The mixing of the grouts was carried out using an EOS-200SH high-speed shear mixer (manufactured in Shanghai City, China), which maintains a constant mixing speed, with a maximum mixing viscosity of 80,000 mPa·s and a maximum rotation speed of 2000 r/min. In this study, the mixing speed was set to a constant 1500 r/min. According to the predetermined experimental proportions, all materials (cement, waste glass, water, or NaOH solution) were weighed and prepared. For the cement grout tests, water was added to the mixing container, the mixer was started, and cement was quickly poured in for thorough mixing. The mixing time was set to 300 s. For the WGP–cement-based grout mixing, water (or NaOH solution) was first poured into the mixing container, the mixer was started, and WGP was gradually added and mixed thoroughly for 300 s. After a WGP grout formed, cement was added and mixing continued for another 300 s before stopping. Once the grout mixing was completed, performance tests were conducted according to the experimental design plan.

### 2.4. Testing Methods

The performance tests of the grouts included tests of their stability and rheological properties, with the experimental procedure outlined in Figure 1. When testing the stability of cement grouts, water separation testing is commonly used [5,8]. This test measures the change in the volume of free liquid in the grout over time, calculates the water separation rate, and evaluates the relative stability of the grout. This method is applicable to various particulate grouts. In this study, the stability of the grout was reflected by the water separation rate of 100 mL of grout (the percentage of free liquid volume to the total grout volume), which indicates the content of free liquid in the cement grout. A smaller volume of free liquid indicates better grout stability. The average free liquid separation rate from five parallel tests was taken as the stability test result for the same mix proportion of grout.

Rheological property testing often employs the rotational viscometer method [64] to measure the shear rate and shear stress of cement, from which a series of rheological parameters (such as apparent viscosity, absolute viscosity, plastic viscosity, shear yield strength, dynamic shear stress, static shear stress, etc.) can be calculated. These parameters can be used to assess the viscosity, rheology, and thixotropy of the grout. In this study, a ZNN-12D digital viscometer (manufactured in Qingdao City, China) with a measurement range of 0 to 1200 mPa·s and a variable speed range of 1, 2, 3, 6, 10, 20, 30, 60, 100, 200, 300, and 600 r/min was used. Rheological curves were plotted based on multiple measurement points to determine the rheological model of the grout and establish the various rheological parameters. It is worth noting that the shear stress at a given rotation speed was taken as the stable reading value after 10 s. If the reading was unstable, the test value was determined when the fluctuation within 30 s did not exceed 2 mPa·s. The average reading from two parallel tests was taken as the viscosity test value for the grout at that mix proportion. If the difference between the two tests was greater than 5%, another batch of grout was prepared and tested again.

## 3. Results and Discussion

### 3.1. Stability of WGP–Cement-Based Grout

**Figure 5:**
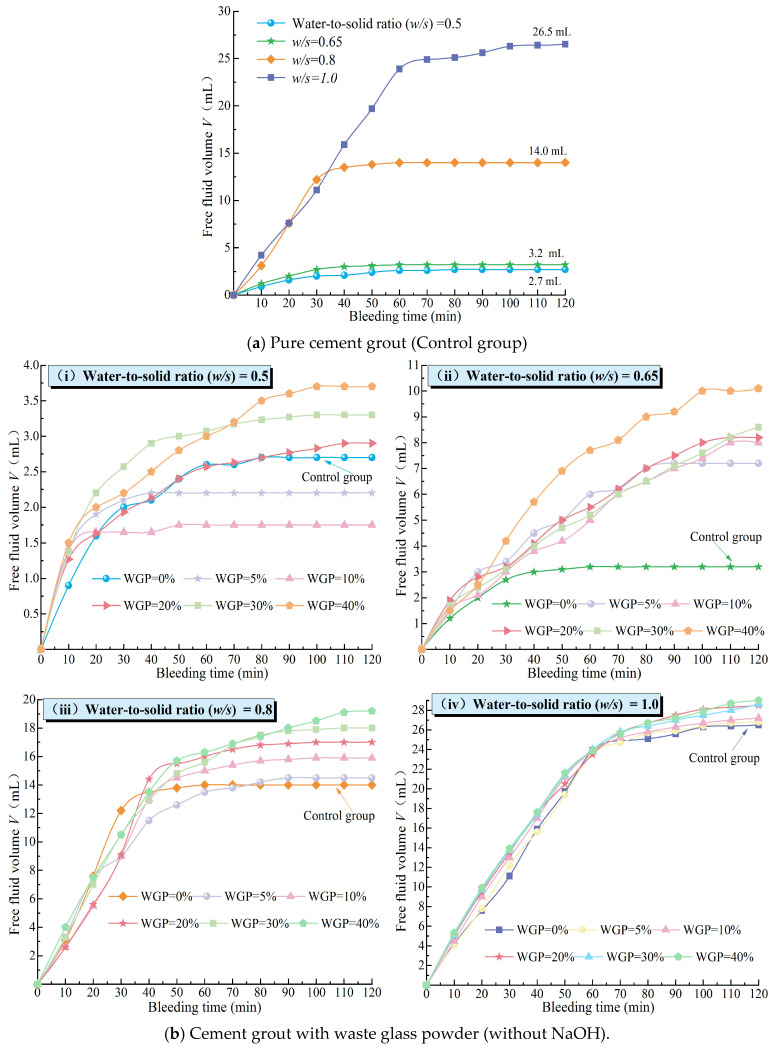

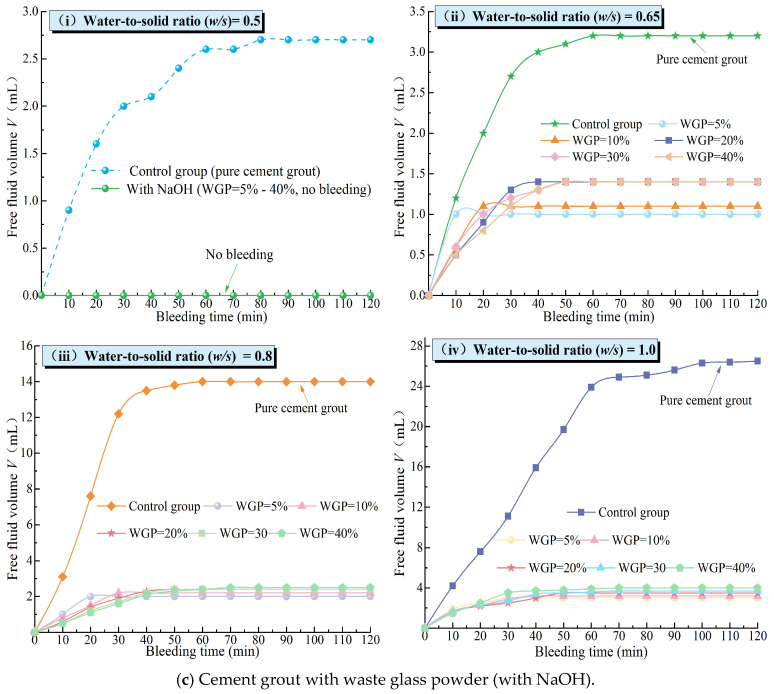
Curves of bleeding volume from grout over time under different mixing ratios.

**Figure 6:**
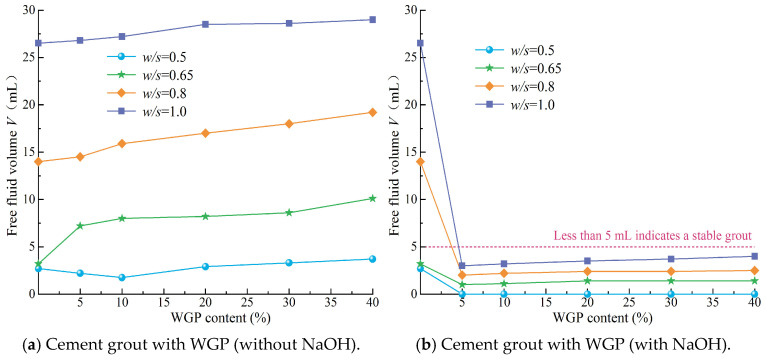
Variation in bleeding volume from grout based on the amount of waste glass powder.

During the stability testing of cement grouts, the volume of free liquid was recorded every 10 min, and the test results were based on five parallel tests. The resulting curves, as shown in Figure 5, depict the semi-“C”-shaped change in free liquid volume over time for different mix proportions. The stable time for the separation of free liquid is defined as the point where the deviation between the volume of free liquid separated at a certain time and the total separated volume is less than 10%, marking the beginning of a horizontal segment in the curve of free liquid volume change over time. As illustrated in Figure 5a, as the water–solid ratio (*w*/*s*) increased, both the separated volume of free liquid and the stable time for its separation gradually increased. This indicates that a higher *w*/*s* ratio results in poorer grout stability and a longer time required to achieve the complete separation of free liquid. Figure 5b and Figure 6a reveal that, in the absence of activators, the volume of free liquid separated from WGP–cement grouts generally increased with the increase in WGP content, suggesting that the incorporation of WGP negatively affects the stability of cement grouts. This is attributed to the smooth surface (Figure 4) and low water absorption rate of waste glass particles (*d*_50_ = 27.55 μm). The incorporation of waste glass reduces the specific surface area of solid particles in the grout (Figure 3), decreasing the amount of water needed to coat the particles and lowering the overall water absorption rate of the solid particles. Consequently, as WGP is added, the amount of excess free liquid increases, leading to an increase in the volume of separated free liquid. This finding aligns with Taha et al.’s [51] research, which reported that the low water absorption and smooth surface of waste glass can cause bleeding and segregation issues. Additionally, it can be observed that for cement grouts with a *w*/*c* ratio of 0.5, the volume of separated free liquid first decreased and then increased with the increase in WGP content, reaching a minimum of 1.75 mL at a WGP content of 10%. This is because, in low *w*/*c* ratio grouts, the free liquid content is relatively low. Although the incorporation of waste glass increases the free liquid content to some extent, this change is relatively small. Furthermore, due to the angular and irregular shape of waste glass particles, non-spherical particles with more edges and corners are more likely to form interconnected structures in the grout, thereby enhancing its stability. However, this stability may hinder the separation of free liquid, as the stable structure obstructs liquid migration and separation. Therefore, this obstructive effect outweighs the enhanced separation effect caused by the increased volume of free liquid due to the low-dose incorporation of waste glass, resulting in a decrease in the separated volume for grouts with a *w*/*s* ratio of 0.5. As the waste glass content increases, the stabilizing effect of waste glass becomes less significant than the separation effect caused by the increase in free liquid volume, leading to an increase in the volume of separated free liquid. Figure 5b shows that the incorporation of waste glass significantly increases the period of stability for the separation of free liquid in grouts with different *w*/s ratios compared to plain cement grouts, indicating that the stabilizing effect delays the separation of free liquid.

In grouting engineering practice, grouts that release less than 5% of their volume as free liquid within 2 h are often referred to as stable grouts. Grouts with high water separation have weak suspension capabilities for solid particles, resulting in poor stability and injectability. Grouts with significant water loss exhibit poor stability, and when they move through the ground, the pressure filtration effect of the pores and fractures in the formation causes excessive water loss, significantly increasing the grout’s viscosity and reducing its fluidity. This can lead to “early setting” phenomena, which, in turn, block the flow path increase in the grout, resulting in flow resistance, and prevent the grout from further diffusing to the designed location within the formation. As a result, the degree of filling the pores and fractures in the formation decreases, and the grouting often fails to achieve the expected results.

It is noteworthy that when WGP is added to cement grout with a water–solid ratio (*w*/s) of 0.65 without an alkaline activator, the volume of free liquid separated from each 100 mL of grout increases from 3.2 mL (3.2%) to 7.2–10.1 mL (7.2–10.1%). This transformation indicates that the grout changes from a stable grout to an unstable one (Figure 6a). For cement grout with a *w*/s ratio of 0.5, the volume of free liquid separated from each 100 mL of grout increases from 2.7 mL (2.7%) to a maximum of 3.7 mL (3.7%) after the incorporation of WGP. This means that when the waste glass content does not exceed 40%, the waste glass–cement grout with a *w*/*s* ratio of 0.5 remains stable (Figure 6a). The performance test results of multi-component mixed grouts incorporating WGP reported in references [65,66] also indicate that WGP has a significant impact on the fresh properties of the slurry, and the stability of the waste glass powder–cement grout increases with the addition of WGP. These findings are similar to those of the present study.

Under NaOH alkali activation conditions, the separation of free liquid from waste glass–cement grouts with different water–solid ratios *(w*/*s*) is significantly reduced compared to that of plain cement paste (Figure 5c and Figure 6b). In particular, the alkali-activated waste glass–cement grout with a *w*/*s* ratio of 0.5 exhibits no separation (Figure 5c), and the stabilization time for separation is shorter than that of plain cement paste. This is contrary to the behavior observed in waste glass–cement grouts without alkali activators. Additionally, under the same *w*/*s* ratio, the variation in separation volume among grouts with different waste glass content is small, showing only a gradual increase. This indicates that increasing the amount of WGP has a minimal impact on the stability of cement grout under alkali activation conditions. For waste glass–cement grouts with a *w*/*s* ratio below 1.0 under alkali activation, the separation volume is less than 5 mL (5%) (Figure 6b). This means that the addition of alkali transforms the unstable grouts with *w*/*s* ratios of 0.8 and 1.0 into stable grouts. This may be due to the reaction between alkaline substances in NaOH and compounds such as calcium silicate and calcium aluminate in cement, which generates hydroxides, carbonates, and other compounds. These newly formed compounds do not enhance the strength of the cement but may affect water separation by altering the chemical composition and properties of the grout. WGP itself possesses a certain degree of pozzolanic activity, although it is relatively low. However, in an alkaline environment, it is activated, allowing it to react with cement components and form more stable hydration products [67]. This may also contribute to the reduction in water separation, making the grout more viscous or cohesive and thereby reducing the free movement and separation of free liquid.

### 3.2. Rheological Performance of WGP–Cement-Based Grout

Under certain conditions, the property of a fluid that allows it to flow and deform under the action of external forces is known as rheology. The shear stress of a fluid is the force per unit area in the direction of the applied force, while the shear rate refers to the change in fluid strain per unit time. Rheology describes the relationship between shear stress and shear rate. The curve representing the relationship between shear stress and shear rate is called the rheological curve, and the equation relating shear stress to shear rate is known as the rheological constitutive equation. Cement-based grouts can be classified into different models based on their rheological constitutive equations, including the Bingham model, the Power-law Model, the Herschel–Bulkley (H-B) Model, and the Carson Model [49,50]. In this study, a rotational viscometer with instrument constants C_1_ = 0.511 and C_2_ = 1.703 is used. The shear stress τ is calculated as *τ* = C_1_ × the reading of the rotational viscometer (Pa), and the shear rate γ is calculated as *γ* = C_2_ × the rotational speed of the viscometer (s^−1^).

**Figure 7:**
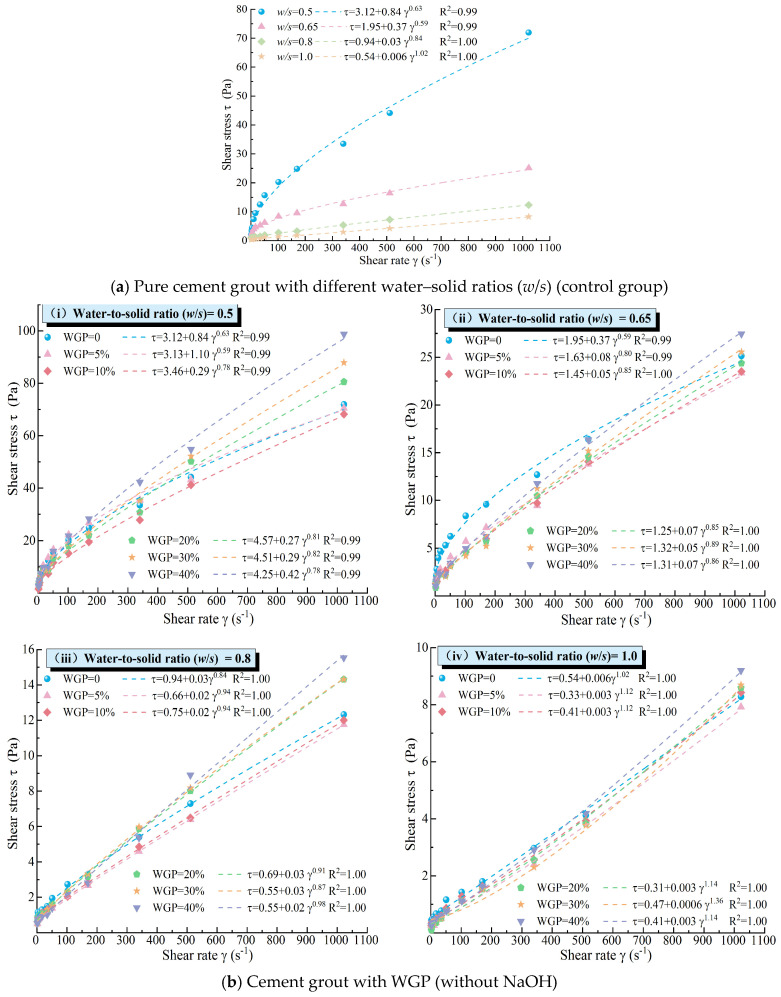

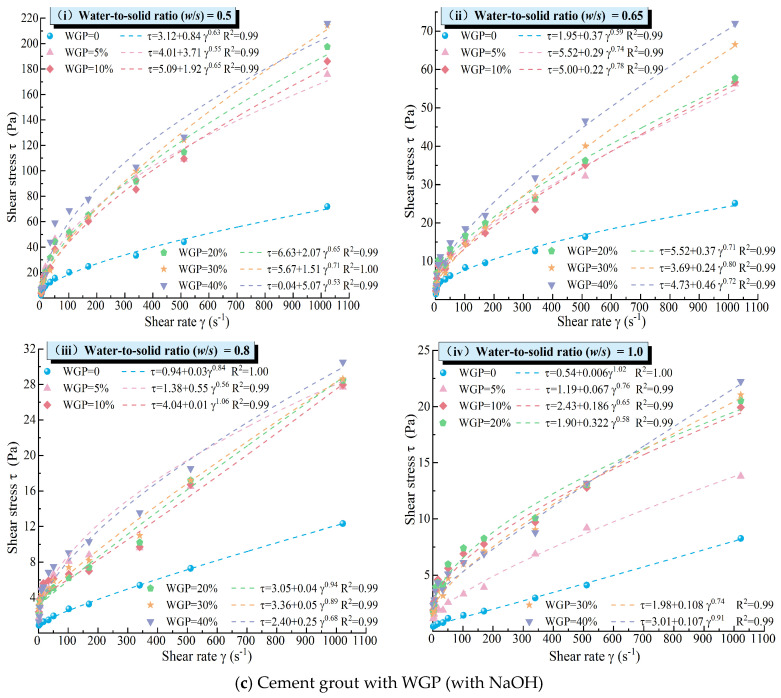
Optimal rheological equations for grout under different conditions.

Based on the test results, the shear rates and corresponding shear stresses of the grouts under different mixing ratios were calculated and plotted as scatter data. Fitting analyses using the Bingham model, Power-law Model, Herschel–Bulkley (H-B) Model, and Carson Model were conducted to determine the optimal rheological model. As shown in Figure 7a, the optimal rheological model for plain cement paste, obtained through fitting analysis, is the H-B model (τ = τ_0_ + k *γ^n^*), where τ is the shear stress (in Pa), τ_0_ is the initial yield stress (in Pa), representing the minimum shear stress required for fluid flow, *k* is the consistency coefficient (in Pa·s^n^), related to the fluid’s viscosity, *γ* is the shear rate, and *n* is the flow behavior index, describing the relationship between shear stress and shear rate. When *n* = 1, the fluid exhibits Newtonian behavior, with shear stress proportional to shear rate. When *n* < 1, the fluid is pseudoplastic, meaning that shear stress increases more slowly as the shear rate increases. The H-B model showed an excellent fit to the rheological performance test data for cement grouts with different *w*/*s* ratios, with R^2^ values exceeding 0.99 for all data, indicating that the H-B model can very accurately explain and predict the variations in the data. Specifically, for the grout with a *w*/*s* ratio of 1.0, *n* ≈ 1, indicating Bingham fluid behavior. For grouts with *w*/*s* ratios of 0.5, 0.65, and 0.8, *n* < 1, indicating pseudoplastic fluid behavior. Therefore, the H-B model was used to fit and analyze the rheological test data for cement grouts containing WGP, and the results are shown in Figure 7. Clearly, the H-B model provided a good fit for the rheological behavior of waste glass–cement grouts under different conditions, indicating that the H-B model effectively characterizes the rheological properties of these grouts. The research conducted by Yin et al. [66] on the rheological properties of cement-based grouting materials containing WGP, water-reducing admixture, and viscosity-modifying admixture (VMA) revealed that the slurry incorporating WGP conforms to the Herschel–Bulkley rheological model and exhibits a significant shear-thickening response. These findings are consistent with those of the present study. Similar conclusions have also been drawn from research on cement-based composites reported in references [68,69].

In the absence of alkali activators, the rheological curves of the grouts with the same water-to-cement ratio did not show significant differences compared to plain cement paste, indicating that the incorporation of waste glass did not alter the flow pattern of the cement-based grouts. The addition of WGP had an impact on the shear stress of waste glass–cement grouts with different *w*/*s* ratios, and the effect was more pronounced at lower *w*/*s* ratios (Figure 7b). A lower *w*/*s* ratio implies a higher concentration of solid particles in the grout, leading to stronger interactions between the particles. Therefore, the influence of WGP on the rheological properties of the grout was more significant at lower *w*/*s* ratios. Notably, for grouts with a *w*/*s* ratio of 1.0, there was evident shear thickening behavior in response to differences in WGP content, with flow behavior indices (*n*) significantly greater than 1.0, indicating pronounced shear thickening. This may be due to the high free liquid content in grouts with high *w*/*s* ratios. Under high shear rates, irregularly shaped and angular waste glass particles move within the paste and undergo significant disordered collisions and mutual constraints with surrounding cement gel and waste glass particles, leading to changes in frictional and contact forces between the particles and affecting the flow behavior of the grout, resulting in noticeable shear thickening during shearing. At lower *w*/*s* ratios, the higher concentration of solid particles in the grout and stronger interactions between the particles result in strong constraints on the disordered motion of WGP by surrounding waste glass particles and cement gel under high shear, thus preventing shear thickening. This also contributes to the more significant effect of waste glass incorporation on the rheological properties of the grout at lower *w*/*s* ratios (*w*/*s =* 0.5). In summary, the influence of WGP on the rheology of cement grouts includes both physical and chemical effects.

Under the influence of NaOH alkali activator, the flow pattern of waste glass–cement grout remained unchanged compared to plain cement grout, with its rheological characteristic indices (*n*) all remaining less than 1, indicating that grouts with *w*/*s* ratios of 0.5, 0.65, 0.8, and 1.0 exhibited pseudoplastic fluid behavior. The incorporation of waste glass did not alter the flow pattern of the cement-based grout, and the H-B model effectively described the rheological behavior of waste glass–cement grout under alkali activation conditions (see Figure 7c). At the same *w*/*s* ratio, the rheological curves of the grouts showed significant differences, primarily reflected in the fact that following alkali activation, the shear stress of waste glass–cement grout at the same shear rate was significantly higher than that of plain cement paste and waste glass–cement grout without activator (see Figure 7b,c for comparison). Under activator conditions, the rheological curves of waste glass–cement grout were all positioned above those of cement grout with the same water-to-cement ratio, and this difference became more pronounced at lower *w*/*s* ratios, indicating a significant increase in the rotational viscosity of waste glass–cement grout under alkali activation. This phenomenon was attributed to the effective release of the activity of physically activated (ball-milled to a particle size of less than 75 μm) waste glass under further stimulation by NaOH alkali activator. When NaOH solution was mixed with WGP, the 2NaOH + SiO_2_ = Na_2_SiO_3_ + H_2_O reaction occurred, producing sodium silicate (Na_2_SiO_3_) and water. The water solubility of sodium silicate imparted a certain viscosity to the mixed grout and facilitated the rapid participation of waste glass in the hydration reaction of cement, leading to the dissolution and gelation of the surface components of waste glass particles. Consequently, the shear-thickening behavior observed in waste glass–cement grout with a *w*/*s* ratio of 1.0 in the absence of activator disappeared upon the addition of activator, exhibiting rheological characteristics similar to those of cement grout with a low *w*/*s* ratio.

**Figure 8:**
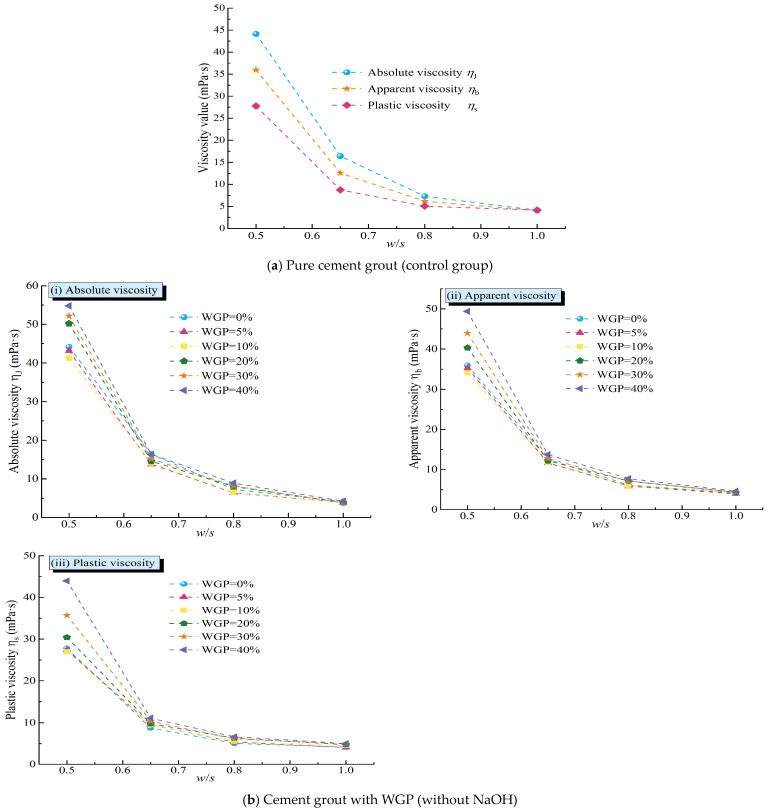

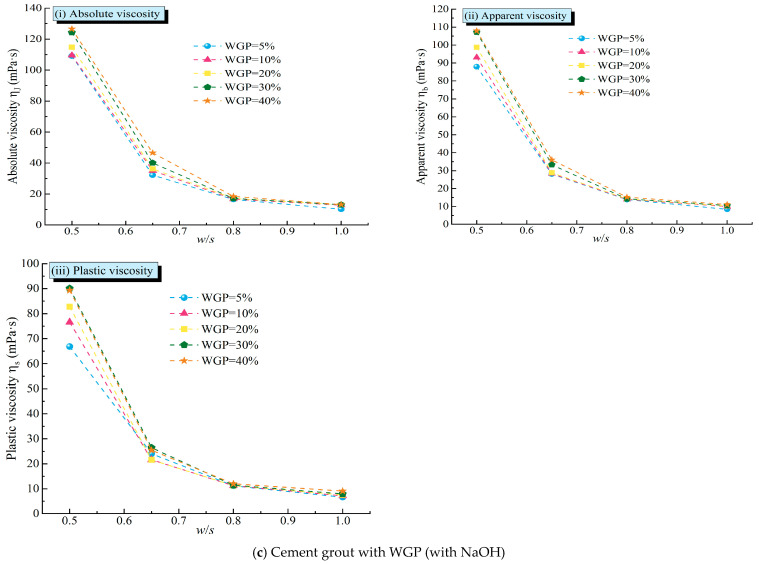
The viscosity of cement grout with different amounts of WGP varies with the water–solid ratio (*w*/*s*).

**Figure 9:**
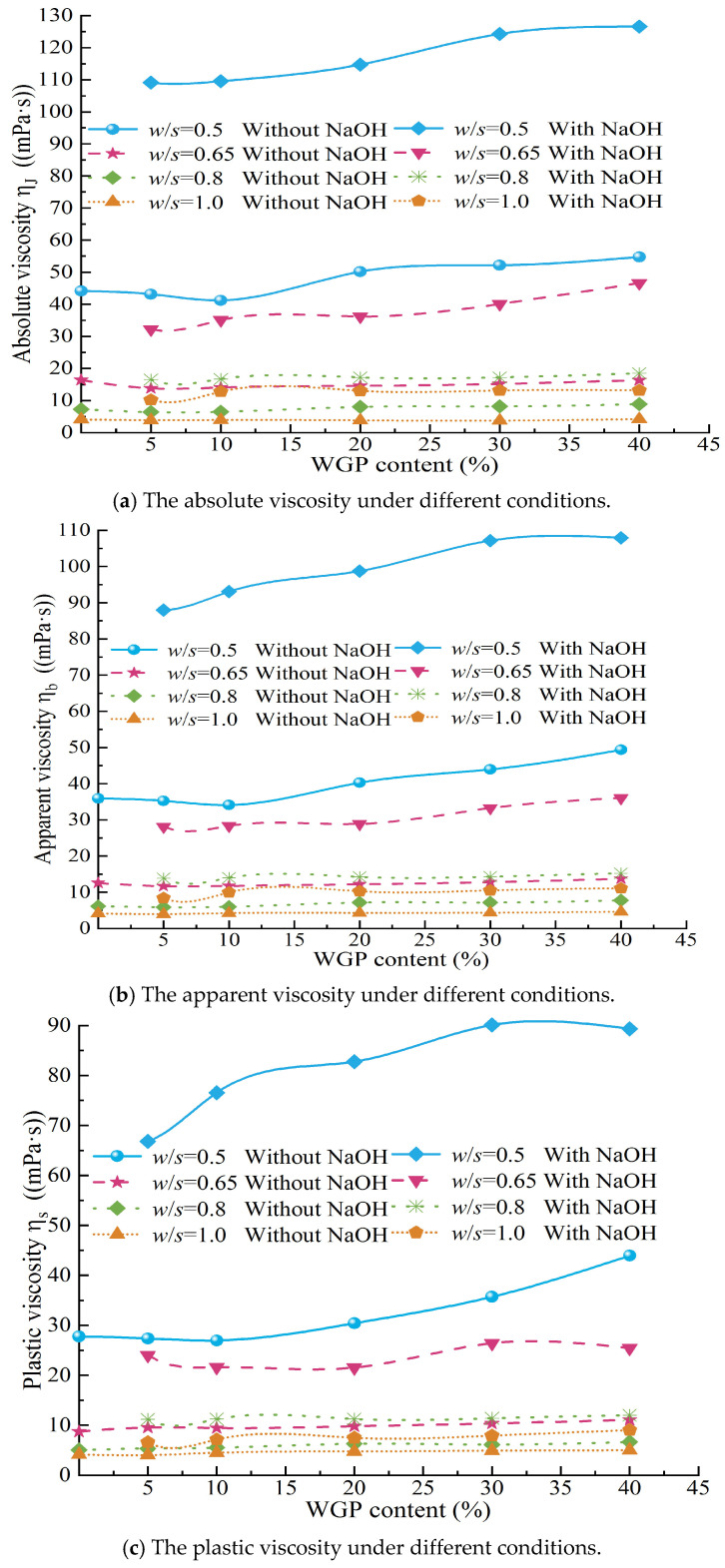
Variation curves of grout viscosity with different amounts of WGP under different conditions.

Rheological tests were conducted on tap water and aqueous solutions of NaOH with *w*/*s* ratios of 0.5, 0.65, 0.8, and 1.0. The results showed that both the NaOH solutions and tap water exhibited Newtonian fluid behavior, and their absolute viscosity (Newtonian viscosity) values were calculated. The absolute viscosity of tap water was found to be 0.8 mPa·s, while the absolute viscosities of the NaOH solutions corresponding to *w*/*s* ratios of 0.5, 0.65, 0.8, and 1.0 were 1.5 mPa·s, 1.2 mPa·s, 1.0 mPa·s, and 0.9 mPa·s, respectively. Clearly, the absolute viscosities of the NaOH solutions were all higher than that of tap water, and as the *w*/*s* ratio increased (i.e., the NaOH content decreased), the absolute viscosity gradually decreased from 1.5 mPa·s at a *w*/*s* ratio of 0.5 to 0.9 mPa·s at a *w*/*s* ratio of 1.0. This indicated that the addition of NaOH increased the viscosity of the solution, and the higher the concentration, the more significant the increase in viscosity. This was one of the reasons for the noticeable increase in rotational viscosity of waste glass–cement grout under activator conditions. However, this increase was relatively limited, with a maximum increase of only 0.7 mPa·s. To further elucidate the effects of alkali activator and WGP content on the viscosity of cement grout, the absolute viscosity (*η*_J_ = *Φ*_300_), apparent viscosity (*η*_b_ = $\frac{1}{2}$ *Φ*_600_), and plastic viscosity (*η*_s_ = *Φ*_600_ − *Φ*_300_) of the grouts under different conditions were calculated based on the rheological test results, as shown in Figure 8 and Figure 9.

As the *w*/*s* ratio increases, the absolute viscosity, apparent viscosity, and plastic viscosity of the neat cement grout (WGP = 0) all exhibit a gradual decreasing trend. Specifically, the rate of decrease in viscosity values with increasing *w*/*s* ratio gradually slows down, which manifests as a gradual flattening of the slope of the line connecting viscosity values at adjacent *w*/*s* ratios. This reveals a transition process from a sharp decrease to a gradual decrease in paste viscosity, as shown in Figure 9a. A similar phenomenon is also observed in Figure 9b,c, where the trends in absolute viscosity, apparent viscosity, and plastic viscosity of cement grouts with WGP, regardless of the addition of alkali activators, remain highly consistent with varying *w*/*s* ratios. Notably, under conditions of high WGP content, the rate of decrease in viscosity with increasing *w*/*s* ratio is significantly higher than that of cement grouts with lower WGP content under the same conditions, especially within the range where the *w*/*s* ratio increases from 0.5 to 0.65. This phenomenon may be attributed to the effective exertion of the structural effect of waste glass in high-consistency grouts, with the structural effect becoming more pronounced as the waste glass content increases. However, as the *w*/*s* ratio further increases, the free liquid content in the grout increases, leading to a gradual weakening of the structural effect of waste glass. As a result, the differences in viscosity among grouts with differing WGP content gradually diminish. On the graphs, this change can be observed based on the viscosity curves for different WGP contents gradually converging as the *w*/*s* ratio increases.

The influence of waste glass powder (WGP) content on the viscosity of cement grout cannot be ignored. Based on the test results, the relationships between the absolute viscosity (Figure 9a), apparent viscosity (Figure 9b), and plastic viscosity (Figure 9c) of the grout under different mixing ratios and WGP content are presented. In the absence of an activator (NaOH), the absolute viscosity, apparent viscosity, and plastic viscosity of grouts with different *w*/*s* ratios all exhibit a trend of first decreasing and then increasing with the increase in WGP content, reaching their minimum values at 10% WGP content. This trend is more pronounced at lower water–solid ratios, meaning that the effect of WGP content on the absolute viscosity is more significant in grouts with low water–solid ratios (*w*/*s* = 0.5, 0.65) without activators, while the absolute viscosity of grouts with high water–solid ratios (*w*/*s* = 0.8, 1.0) shows less variation with WGP content. This may be attributed to the dual effects of WGP in cement grout when no activator is added. Firstly, WGP, with its smooth surface and distinct edges, has a low water demand and can release more free liquid (exhibiting a water-releasing effect). Secondly, as the WGP content increases, the collision and friction between particles under shear action are enhanced (structural effect). The release of free water can reduce the viscosity of the grout to a certain extent, but this reduction is limited. On the other hand, the structural effect of WGP under shear affects the rheology of the grout. The influence of this shear-induced structural effect on the absolute viscosity of cement grout is strongly regulated by the *w*/*s* ratio. The impact of WGP incorporation on viscosity is primarily reflected in its physical effects rather than via chemical reactions. At low *w*/*s* ratios, the grout itself is relatively viscous. When the WGP content is low, the shear-induced structural effect of WGP is weak, and the increase in free water in the grout makes the rheology of the viscous grout more sensitive to this increase. As a result, the viscosity of the grout decreases relatively significantly during the process; this is in agreement with the work of Yin et al. [66], who concluded that WGP has the effect of reducing viscosity. In contrast, at high *w*/*s* ratios, the grout contains high free water content and has good fluidity. The rheology of the grout is less sensitive to the increase in free water, and the high free liquid content makes the particle structural effect during shear less pronounced. Therefore, the influence of WGP content on viscosity is relatively small in this case. This is reflected in Figure 9, where the curves of absolute viscosity, apparent viscosity, and plastic viscosity of grouts with high *w*/*s* ratios show little variation with WGP content, appearing nearly horizontal. These differences highlight the complex mechanism of WGP’s effect on the rheological properties of cement grout and the important influence of the *w*/*s* ratio on grout rheology.

When an alkali activator is added, the incorporation of waste glass not only affects the rheology through physical interactions but also generates chemical reaction effects, significantly influencing the viscosity of the grout (as shown in Figure 9). For waste glass cement pastes with alkali activators, as the WGP content increases, and the absolute viscosity, apparent viscosity, and plastic viscosity gradually increase. In Figure 9, lines of the same color represent the viscosity changes in grouts with and without alkali activators as a function of waste glass content. It is evident that the viscosity-change curves of grouts with alkali activators are located above those of grouts without alkali activators under the same conditions, and this increase is more pronounced at lower *w*/*s* ratios (as demonstrated by the larger vertical distances between the lines). This indicates that under the same *w*/*s* ratio, the viscosity of the grout significantly increases after the addition of an alkali activator, and this increase is more significant at lower *w*/*s* ratios. This also illustrates the complex interaction mechanism between alkali activators, waste glass content, and *w*/*s* ratios on the rheology of the grout. In fact, the viscosity increase in aqueous solutions of NaOH at *w*/*s* ratios of 0.5, 0.65, 0.8, and 1.0 is very limited compared to that of tap water, with a maximum increase of only 0.7 mPa·s. Therefore, after the addition of an alkali activator, the chemical reaction effects between the NaOH, WGP, and cement particles have the greatest impact on the viscosity of the grout.

## 4. Conclusion

(1) WGP incorporation generally increases free liquid separation in grout, reducing stability, especially at high *w*/*s* ratios without an activator. However, at low *w*/*s* ratios (e.g., 0.5) and with 10% WGP, stability is temporarily improved;
(2) At high *w*/*s* ratios (0.65) and without an activator, WGP significantly destabilizes grout, whereas at low *w*/*s* ratios (0.5) and with ≤40% WGP, grout remains stable;
(3) NaOH alkali activation reduces free liquid separation and shortens the stabilization time in waste glass cement grout, stabilizing originally unstable grouts at *w*/*s* ratios < 1.0;
(4) The Herschel–Bulkley model accurately describes waste glass cement grout rheology, with *R*^2^ > 0.99, effectively characterizing rheological properties for both pure and WGP-containing grouts;
(5) WGP and NaOH significantly affect grout rheology, altering shear stress and viscosity, particularly at lower *w*/*s* ratios. Alkali activation increases shear stress and viscosity, with more pronounced effects at lower *w*/*s* ratios;
(6) Grout viscosity is jointly regulated by the *w*/*s* ratio, WGP content, and alkali activator, exhibiting a complex interaction mechanism. As the *w*/*s* ratio increases, viscosity decreases, while WGP content has a non-linear effect on viscosity, and alkali activation further increases viscosity, especially at lower *w*/*s* ratios.

Notably, this study focuses on the stability and rheological properties of slurries prepared by mixing WGP with P.O 42.5 ordinary Portland cement under different *w*/*s* ratios, both with and without the addition of NaOH. Therefore, important areas for future research include the compatibility of WGP with different types of cement, optimization of WGP content and activator dosage, and exploration of more environmentally friendly activators.

## Figures and Tables

**Table 1:** Different combinations of waste glass (WG) with other by-products.

Topic	Usage	Focus	Novelty/Contribution	References
Cement Concrete	Binder; aggregate	Workability; strength; durability	A comprehensive analysis was conducted to determine the characteristics of WG concrete and the challenges faced in the application of WG	[1,2,9,10,11]
	Fine and coarse aggregate	Workability; durability; strength	Concrete with WG exhibits better performance, with particle size influencing workability, while durability-related properties remain consistent	[21,23]
	Fine aggregate	Abrasion and freeze–thaw resistance; sulfate attack; penetration	Concrete with WG exhibits similar properties compared to normal concrete.	[18,19,22,24,40]
Self- compacting concrete	Fine and coarse aggregate	Strengths; absorption; failure characteristics; microstructure	It is possible to produce self-compacting concrete using WG, and concretes containing WG exhibit less brittle behavior than the reference concrete	[15,16]
Barite concrete	Fine aggregate	Workability; mechanical properties; alkali–silica reaction	It is efficient, economical and environmentally benign to use WG to produce high-density radiation shielding concrete	[14]
Steel slag concrete	Coarse aggregate	Slump; density; modulus of elasticity; strength	The ability to improve the fire resistance of concrete	[17]
Geopolymer concrete		Strengths; porosity; sorptivity; chloride permeability	The properties of geopolymer concrete containing 10 to 20% WG were comparable to the corresponding properties of the control	[25]
	Binder	Alkali–silica reaction; durability and sustainability	WG geopolymer concrete is classified as a durable structural material with broad application prospects, serving as a promising construction material	[30,36,37]
Mortars	Binder/ fine aggregate	Alkali–silica reaction; pore solution	WGP with a particle size of less than 300 μm exhibits an excellent mitigation effect on ASR expansion.	[20,28,29]
Alkali- activated mortars	Fine aggregate	Workability; strength; drying shrinkage; chloride permeability; et al.	In alkali-activated mortar, replacing natural sand with WG cullet in different proportions can result in a performance comparable to that of natural sand	[32]
	Binder	Workability; strength; permeability; microstructure; et al.	The feasibility of using WGP as a partial precursor in alkali-activated mortars was investigated	[33,35,38]
Cement- based grout	Binder (*w*/*s* = 1.3)	The Vicat; compressive strength; ultrasonic pulse velocity	The use of cement-based grout combined with WGP to enhance the clay soil via a deep mixing technique	[12]
	Binder (*w*/*s* = 0.5)	Electrical resistivity and strength; shrinkage	The properties of binary and ternary cement pastes containing WGP were examined	[27]
Geopoly- mer grout	Binder (*w*/*s* = 0.6)	Compressive strength; microstructure	The obtained compressive strengths were about 73–104% of those of the control cement-based grout	[13]
	Binder (*w*/*s* = 0.42)	Compressive strength; microstructure	This work significantly advances the understanding of the reactivity and potential of WGP	[34]
Cement- based grout	Binder (*w*/*s* = 0.5, 0.65, 0.8, 1.0)	Stability and rheology	This work studies the influence of WGP content and NaOH on the stability and rheological properties of grouts at different *w*/*s* ratios	This study

**Table 2:** Partial performance parameters of P.O 42.5 Portland cement.

	Specific Surface Area (m^2^/kg)	Loss on Ignition (%)	Slag Content (%)	Water-Based Grinding Aid (‰)	Initial Setting Time (min)	Final Setting Time (min)	Alkali Content (%)	Soundness
P.O 42.5	362	3.38	11.5	1.0	158	290	0.38	Qualified
Standard	≥300	≤5.0	5.0~20.0	≤5	≥45	≤600	0.6	Qualified

**Table 3:** The chemical compositions of cement and WGP (%).

Constituent	SiO_2_	CaO	AL_2_O_3_	Fe_2_O_3_	K_2_O	MgO	SO_3_	Na_2_O	Others
Cement	21.40	62.40	6.32	4.15	0.62	2.30	2.40	0.1	0.31
WGP	70.35	10.89	1.85	0.89	0.69	1.29	0.22	12.72	1.0

**Table 4:** Mixture proportions of the grout.

Items	*w*/*s*	Cement Content	WGP Content	Activator Content (Na_2_O)
Control group	0.5 0.65 0.8 1.0	100% 95% 90% 80% 70% 60%	0% 5% 10% 20% 30% 40%	0%
Without activator				
With activator				4%

## Data Availability

Some or all data, models, or code that support the findings of this study are available from the corresponding author upon reasonable request.
